# Genotype profiles of high-risk human papillomavirus in women of reproductive age: A community-based study

**DOI:** 10.1371/journal.pone.0287399

**Published:** 2023-07-17

**Authors:** Dewi Wulandari, Reiva Wisdharilla Meidyandra, Andrijono

**Affiliations:** 1 Department of Clinical Pathology, Faculty of Medicine, Universitas Indonesia/Cipto Mangunkusumo General Hospital, Jakarta, Indonesia; 2 Department of Obstetrics and Gynecology, Faculty of Medicine, Universitas Indonesia/Cipto Mangunkusumo General Hospital, Jakarta, Indonesia; University Federico II of Naples, ITALY

## Abstract

**Purpose:**

This research is a preliminary study to observe the high-risk HPV infection profile among asymptomatic women, as a basis for developing Indonesian-specific reagents and implementing a national vaccination program.

**Methods:**

The research subjects were female employees/families of BUMN (state-owned enterprises) who underwent annual routine medical check-up. The research sample was a cervical swab which was examined using the Cobas^®^ 6800 platform for HPV identification and INNO Lipa (Fujirebio) kit for identification of the genotype.

**Results:**

Out of 858 cervical swab samples collected in this study, 31 samples were excluded as they had invalid results from the initial examination, so the remaining 827 samples continued this study protocol. Of those samples, 69 (8%) samples gave positive results, giving an overall HPV prevalence of 8%. Among HPV-positive samples, HPV 52 was the most common genotype (N = 15, 21.7%) found as both single-infection and multiple infections. The median age of subjects was 38 years. There were nine samples (13%) with multiple infections of two or more genotypes and seven samples (10.1%) with no specific genotype identified.

**Conclusion:**

HPV prevalence was 8%, with HPV 52 being the most common high-risk type, making it a necessity to develop a diagnostic kit and vaccine for national vaccination program that is specific for Indonesian population which includes this genotype.

## Background

Cervical cancer is the fourth most common malignancy in women worldwide and remains as a global health problem. Based on data from the World Health Organization (WHO), in 2020 there are approximately 604,000 new cases and 342,000 deaths from cervical cancer worldwide, and around 90% of them came from low-middle-income countries [1]. In general, globally it is estimated to range from 75 per 100,000 women to 10 per 100,000 women [2]. In Indonesia, based on 2012 data, there were 20,928 recorded cases with 9,498 deaths associated with cervical cancer.

Nonetheless, cervical cancer can be prevented. As Human Papillomavirus (HPV) is found in >95% of cervical cancer lesions, HPV infection is considered an important etiological factor. There is no treatment for the virus itself, although some topical microbicides have been proposed for an intrinsic antiviral activity [3]. Although most HPV infections are asymptomatic and can be self-limiting, all women have a risk of persistent HPV infection which can develop into precancerous lesions that can lead to cervical cancer [4]. High-risk HPV, such as HPV 16 and 18, are considered responsible for cervical low- and high-grade squamous intraepithelial lesions (LSIL and HSIL, respectively). However, if the precancerous lesion is found at an early stage, the lesion can be treated and removed [5]. This entire pathogenesis can be prevented by vaccination, which protects individuals from being infected by HPV in the first place [6]. This is proven in developed countries, where the incidence decreased after implementing HPV vaccination programs for adolescent girls, and regular screening for adult women. Regular screening allows pre-cancerous lesions to be identified at all stages, which can be managed promptly and adequately. In low-middle-income countries, preventive facilities and access are still limited, so most patients only seek help from health facilities when the disease was already at an advanced stage [1]. Similar situation is commonly seen in Indonesia, where around 66.4% of patients got diagnosed with stage IIB-IVB [5].

Human Papillomavirus (HPV) belongs to the Papillomaviridae family. Based on various genomic studies, more than 200 genotypes have been identified. HPV is able to infect the stratum basalis squamous epithelium of the skin and mucous membrane, so it can be classified into cutaneous and mucosal types. In addition, based on its relationship with cancer and pre-cancerous lesions, HPV is also grouped into high-risk types associated with malignant lesions, and low-risk types associated with benign lesions [6].

HPV infection is one of the most common sexually transmitted infections in both men and women. Global prevalence rates of genital HPV infection in men range from 3.5 to 45% compared to 2–44% in women, with similar transmission rates [7]. Most cases of HPV infection in men don’t cause any symptoms, and the infection commonly resolves on its own. Genital warts are the most common clinical manifestation of symptomatic HPV infection in men, which are benign and not associated with mortality. Many women get cervical HPV infections, and most do not progress to cervical cancer [8]. The integration of HPV into the cell genome is considered one of the main factors for cervical cancer development. HPV symptoms may not begin to appear months or years after infection, which makes it tough to detect until genital warts or cancerous lesions start to develop. Detection is even more difficult in women, where the site of infection is anatomically invisible; which is why the program in this study focused on the women population.

HIV-infected individuals are reported to have a higher, up to six times the risk of contracting HPV. However, most infections among immunocompetent individuals will undergo spontaneous elimination. Persistent infections are thought to be associated with neoplastic progression. Persistent low-risk infection can cause benign lesions, such as condyloma, while persistent high-risk infection can cause precancerous lesions, such as CIN (cervical intraepithelial neoplasia) in cervical tissues, which subsequently may develop into invasive cancer [6, 9].

As an effort to eliminate cervical cancer, the WHO mandated all countries by 2030 to have achieved the 90-70-90 target, namely: 90% of young girls to have received full vaccination at 15 years old, 70% of women to have undergone screening using high-performance tests at 35 years old and a repeat testing at 45 years old, and for 90% of women with cervical cancer to be managed (90% of women with pre-cancerous lesions to have fully recovered, and 90% of invasive cancer cases to having been treated) [2]. Indonesia responded to this target by planning a HPV vaccination program for female teens as one of the national vaccination programs [10]. However, this program has yet to cover the adult women population. In line with the government’s program to develop its domestic diagnostic modalities, and to expand the national scope of screening reach, it is necessary to create diagnostic reagents that are easy and cost-effective, while simultaneously being sensitive and specific for genotypes commonly discovered among Indonesian population. For this purpose, as the basis for the development of Indonesia-specific reagents, it is necessary to conduct a preliminary study to identify the high-risk genotypes that are often found in the Indonesian population. This research is a preliminary study to determine the high-risk HPV profile in women of reproductive age.

## Method

### Setting and participants

This cross-sectional, community-based study was conducted between May–December 2022 as a part of a research for diagnostic kit development project. All subjects were female employees and their families belonging to state-owned enterprises (BUMN) in areas of Jakarta, Bogor, Bandung, or Semarang cities who underwent routine medical check-ups and were subjected to a Pap test with no apparent clinical symptoms. A total of 858 subjects participated in this study.

### Samples collection

The sample used in this study was a cervical swab, which was taken by trained health workers using a cytobrush and promptly preserved in a liquid transport medium (ThinPrep PreservCyt Solution^®^—Hologic, Inc. Malborough, MA, USA). The sample was immediately sent to the Immunology and Biomolecular Laboratory, Clinical Laboratory Department, Faculty of Medicine Universitas Indonesia, Cipto Mangunkusumo Hospital, Jakarta.

### HPV identification

HPV identification was carried out using Cobas^®^ HPV from Roche diagnostics using the Cobas 6800^®^ kit, which detects HPV 16, 18, and other high-risk types reported as "others". An amount of 1 mL of liquid transport medium was transferred into a secondary tube suitable for the Cobas 6800^®^ system. Afterwards, the examination was carried out according to the Cobas 6800^®^ standard operating procedure. Positive and negative controls were included in each running batch.

### HPV genotyping

Detection of HPV in each sample was followed by identification of the genotype using INNO-LiPA HPV Genotyping Extra II (Fujirebio, Gent, Belgium), designed for the identification of 32 different genotypes of HPV (16, 18, 31, 33, 35, 39, 45, 51, 52, 56, 58, 59, 68, 26, 53, 66, 70, 73, 82, 6, 11, 40, 42, 43, 44, 54, 61, 62, 67, 81, 83, 89), including 13 HR types on 65 bp fragments. This kit amplifies a 65 bp long region on the open reading frame L1, producing a biotin-labeled amplicon. Genotype detection was based on reverse hybridization. Amplicons were reacted with various genotype-specific oligonucleotide probes attached parallel to the nitrocellulose membrane strips. Subsequently, the conjugate was added in the form of streptavidin-alkaline phosphatase to bind to the biotin-labeled amplicon, which was bound to the probe. The addition of the BCIP/NBT chromogen substrate formed a colored band on the strip.

### Statistical analysis

Data were analyzed using Microsoft Excel 2018 (version 16.16.14 or higher). Categorical variables were summarized using absolute values and percentages. The normality of the distribution of continuous variables was tested by Kolmogorov-Smirnov (K-S) test. Continuous variables with normal distribution were presented as mean (standard deviation [SD]), and non-normal variables were reported as median (interquartile range [IQR]). The difference in age between HPV-positive subjects and HPV-negative subjects was analyzed using the Mann-Whitney U test. Statistical analyses were performed using Statistical Package for Social Science (SPSS) software version 23 (IBM Corporation, Armonk, NY, USA). A p-value (two-tailed) <0.05 was considered statistically significant.

### Ethics approval

This study was approved by the Ethical Clearance Committee of the Faculty of Medicine, University of Indonesia (No. 674/UN2.F1/ETIK/PPM.00.02/2022). The study was conducted in accordance with the Declaration of Helsinki. Written informed consent was obtained from subjects before enrolment.

## Results

Out of 858 cervical swab samples collected in this study, there were 31 samples excluded as they had invalid results from the initial examination, so the remaining 827 samples continued this study protocol. Of those samples, 69 (8%) samples gave positive results. Based on the K-S test, data distribution for age variables does not follow a normal distribution (p<0.05). Therefore, we presented the age variable as median (IQR). The median age of HPV-negative subjects was 38.0 years (IQR: 31–43 years), which was not significantly different from the HPV-infected subjects whose median age was 38.0 (IQR: 32–42 years), with Mann-Whitney p-value of 0.841.

**Fig 1:**
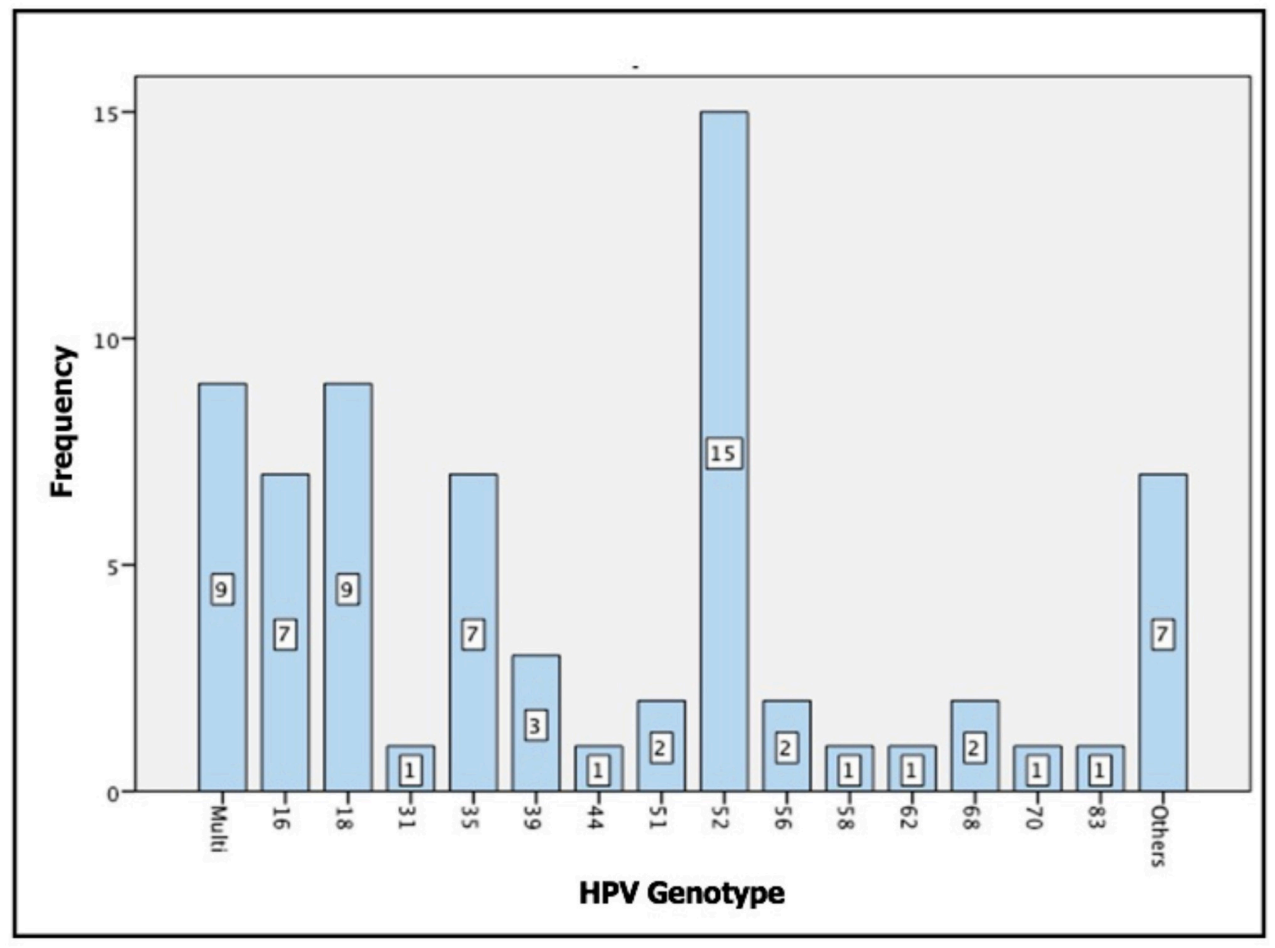
Frequencies of each HPV genotype.

Fourteen genotypes were identified in this study (Table 1), and there were 7 HPV-positive samples with no genotype identified; while 9 samples were identified with multiple genotype infections. HPV 52 was found to be the most frequent high-risk type, both as a single infection and multiple infections (Fig 1).

**Table 1:** Frequencies of each identified HPV genotype.

HPV Genotype	n (%)	Type
16	7 (10,1)	HR
18	9 (13,0)	HR
31	1 (1,4)	HR
35	7 (10,1)	HR
39	3 (4,3)	HR
44	1 (1,4)	LR
51	2 (2,9)	HR
52	15 (21,7)	HR
56	2 (2,9)	HR
58	1 (1,4)	HR
62	1 (1,4)	
68	2 (2,9)	pHR
70	1 (1,4)	pHR
83	1 (1,4)	
Others	7 (10,1)	
Multiple	9 (13,0)	

*HR: high risk; LR: low risk; pHR: probable high risk

Multiple infections occurred due to coinfection of two or more HPV genotypes, either high-risk types or coinfection with low-risk types. HPV 52 was the most frequently found high-risk type in coinfection with other high-risk types, such as 35, 16, and others. Coinfection of high-risk and low-risk types occurred in 1 sample, which was coinfected by HPV 59 and 44. There was one patient who was coinfected by 4 types of HR (35, 52, 58, 70).

## Discussion

Until 2021, more than 200 HPV genotypes have been identified. Based on their ability to trigger the development of precancerous lesions, these HPV genotypes are grouped into high-risk types associated with the risk of precancerous lesions, and low-risk types associated with benign lesions. High-risk types have been widely reported in at least 13 genotypes (16, 18, 31, 33, 35, 39, 45, 51, 52, 56, 58, 59, 68), but HPV 16 and 18 are considered the most prevalent. However, the distribution of commonly found genotypes can vary, depending on geographical areas and different cultural characteristics [11–13]. This study included women of asymptomatic female employees and their families belonging to state-owned enterprises (BUMN) in areas of Jakarta, Bogor, Bandung, or Semarang, representing women of mid to high-social economy and education status living in urban communities.

In this study, the prevalence of HPV infection in the asymptomatic female population was found to be 8%. This is similar to de Sanjosé’s previous report, which stated that the average prevalence of HPV in 13 European countries infection in women with normal cervical cytology was 6.6%, ranging between 1.4–25.6% [14]. Likewise, the meta-analysis conducted by Bruni et al.—which reported data from 5 continents—found that the prevalence of HPV infection in Southeast Asia (including Vietnam, Thailand, and Indonesia) in the female population with normal cervical cytology was 8.4% (95% CI 7.6–9.2) [15].

In this study, the most common high-risk genotype was HPV 52, both as single and multiple infections. These results are different from other studies, which generally reported HPV 16 as the most common high-risk type [15, 16]. This needs to be taken into consideration upon planning a national vaccination program.

Because the commercial HPV vaccines in the circulation generally incorporate HPV 16 as the main target, such as monovalent (HPV16), divalent (HPV16/18), and quadrivalent (HPV 6/16/18/11) vaccines. While the vaccine might be able to prevent infection against HPV 16 and 18 (divalent and quadrivalent), it is not enough to protect against other high-risk types [12]. The same is true in selecting reagent kits for screening tests. In general, the reagents in circulation mostly feature HPV 16 and 18 as the main targets, plus a mixture of various other high-risk types without specifying them.

There is already a nonavalent vaccine commercially available, which protects against HPV 52 among eight other high-risk genotypes (6/11/16/18/31/33/45 and 58), but the vaccine routinely used in Indonesia’s HPV program is the quadrivalent type, which only covers against HPV 6, HPV 11, HPV 16, and HPV 18. By adding HPV 31/33/45/52 and 58 to the genotypes already included in the quadrivalent vaccines, up to 90% of high-grade cervical lesions can be prevented. Using high-estimate calculations, Capra et al. demonstrated that the nonavalent HPV vaccine has a significant additional impact on both LSIL and HSIL, with a 23.8–32.8% increase in the proportion of cases effectively prevented by the second-generation HPV vaccine (absolute additional impact) [4]. Result of the current study is important to offer insight and evidence about the high prevalence of cancer-causing HPV 52 among Indonesian women population, which mandates the need for vaccination that does protect against it.

Another data found in this study was that 10.1% of samples were positive for HPV with an undeterminable genotype. This was possible because reagent kits generally used primers designed to amplify the most conserved DNA sequences and covered most of the HPV genotypes. The region considered to be the most conserved in the HPV genome is the L1 region. Genotype detection was then carried out by adding specific oligonucleotide probes for each known genotype. In this case, the failure to identify the HPV genotype was due to the limitations of the oligonucleotide probes used in the reagent kit.

HPV is generally transmitted through sexual contact, but some suggest that HPV can also live in the environment, as HPV is a stable virus and can survive on fomites and surfaces for days. However, much work is needed to confirm the non-sexual transmission of HPV [17, 18]. Most HPV infections (70–80%) will resolve spontaneously in less than 2 years, but about 20–30% will persist, and 1–2% of them will develop into cervical cancer. Several factors play a role in the persistence of this infection, including the genotype of the virus, multiple infections, the immunological status of the host, personal hygiene, smoking, and alcohol consumption [11]. The median age of the HPV-positive subjects in this study was 38.0 years (IQR: 32–42 years), similar to the HPV-negative subjects (median 38.0 years, CIR: 31–43 years). This finding was slightly different from another study by Zhao et al, which reported that most HPV infections in their study occurred in women aged 40–49 years (>44%) [16]. It may be caused by the subjects’ age in our study, which is mostly younger than 40 years old. Our findings were also different from a meta-analysis conducted by Bruni et al., which included studies on five continents, which reported that the largest age group was <25 years, and the prevalence rate was sloping in the middle-aged female population [15].

Even though this research was conducted on a demographic of middle socioeconomic status, HPV infection was still detected in significant proportion. This is consistent with previous reports, which were hardly contested, which stated that at least 80% of sexually active women will acquire HPV. But this is not the only factor that causes precancerous lesions. Persistent infection is an important factor for cervical carcinogenesis, and multiple infections proved to be an important factor in the persistence of infection [11, 19, 20]. Persistence of infection can also occur in low-risk coinfection and recurrent infections, which are related to multiple sexual partners, personal hygiene, and individual immune status, and therefore are closely linked to socio-economic conditions, degree of education, and access to health facilities that allow people to obtain vaccination, screening tests, and early detection, as well as adequate treatment.

## Limitation

This study has several limitations. First, we only include subjects with relatively homogenous demographic characteristics, representing women of mid to high- social economy and education status living in three big cities; therefore, they may not represent general Indonesian women HPV prevalence status, in which population living in rural areas or low economic rate may encounter younger age of marriage (<15 years old) and thus are more vulnerable to HPV infection. Second, we could not determine the genotype of 7 HPV-positive samples due to the limitations of the oligonucleotide probes used in the reagent kit. Whole-genome sequencing may be a preferable method for assigning HPV genotypes. However, these strategies are associated with higher costs, and only variable portions of the genome are informative for HPV genotyping. Third, we did not collect the results of the Pap test, and therefore, the association of cervical cytology with HPV genotypes could not be inferred in this current study.

## Conclusion

The prevalence of HPV infection among the female population in four major cities of Indonesia is 8%, with a median age of 38 years. The most commonly found high-risk genotype was HPV 52, either as a single infection or co-infection.

## Recommendation

It is essential to develop more suitable reagent kits for Indonesian population. Vaccine selection for the national vaccination program needs to consider the high-risk genotype profile with high prevalence in Indonesia.

## Supporting information

S1 Data (XLSX)
